# Electrophysiological mechanisms of long and short QT syndromes

**DOI:** 10.1016/j.ijcha.2016.11.006

**Published:** 2016-11-26

**Authors:** Gary Tse, Yin Wah Fiona Chan, Wendy Keung, Bryan P Yan

**Affiliations:** a Department of Medicine and Therapeutics, Chinese University of Hong Kong, Hong Kong, SAR, PR China; b Department of Psychology, School of Biological Sciences, University of Cambridge, Cambridge, United Kingdom; c Stem Cell & Regenerative Medicine Consortium, Li Ka Shing Faculty of Medicine, The University of Hong Kong, Pokfulam, Hong Kong, SAR, PR China; d Department of Epidemiology and Preventive Medicine, Monash University, Melbourne, VIC, Australia

**Keywords:** Cardiac arrhythmia, Repolarization, Long QT syndrome, Short QT syndrome, Wavelength

## Abstract

The QT interval on the human electrocardiogram is normally in the order of 450 ms, and reflects the summated durations of action potential (AP) depolarization and repolarization of ventricular myocytes. Both prolongation and shortening in the QT interval have been associated with ventricular tachy-arrhythmias, which predispose affected individuals to sudden cardiac death. In this article, the molecular determinants of the AP duration and the causes of long and short QT syndromes (LQTS and SQTS) are explored. This is followed by a review of the recent advances on their arrhythmogenic mechanisms involving reentry and/or triggered activity based on experiments conducted in mouse models. Established and novel clinical risk markers based on the QT interval for the prediction of arrhythmic risk and cardiovascular mortality are presented here. It is concluded by a discussion on strategies for the future rational design of anti-arrhythmic agents.

## 1 Introduction

Long and short QT syndromes (LQTS and SQTS) are primary electrical disorders of the heart that predispose the affected individuals to sudden cardiac death *via* the development of malignant ventricular arrhythmias. Both syndromes can arise congenitally from ion channel mutations, or can have acquired causes. In this article, the ionic basis of the QT interval is examined, summarizing recent advances into the electrophysiological mechanisms of arrhythmogenesis of both LQTS and SQTS.

### 1.1 The QT interval

The QT interval of the human electrocardiogram (ECG) is a marker of the duration of the cellular action potential (AP) [1]. It varies with heart rate, and therefore a correction must be made before its interpretation. Different formulae have been proposed for this purpose (Table 1). The commonest is Bazett's formula, given by the QT interval divided by the square root of the RR interval. However, this method overestimates QT interval at high heart rates and underestimates it at low heart rates [2]. By contrast, Fridericia formula, in which QT interval is divided by the cubic root of the RR interval, works better for slow heart rates. Other methods include the Framingham and Hodges formulae. The upper limit of a normal corrected QT (QT_c_) interval by Bazett's formula is 440 ms for males and 460 ms for females. The latest European Society of Cardiology guideline produced in 2015 suggests upper and lower limits of 480 ms and 360 ms, respectively, for both males and females [3]. The QT interval increases with age and long QT interval is commonly associated with electrolyte abnormalities [4], drugs [5], [6], [7], medical conditions such as epilepsy and diabetes mellitus [8], [9]. The risk of arrhythmogenesis is increased at both extremes of the QT interval. To understanding why this is the case, the ionic determinants of the AP and the mechanisms by which their alterations lead to repolarization abnormalities must be considered.

**Table 1:** Different methods of QT correction.

QT correction method	Formula
Bazett	QT/RR^1/2^
Fridericia	QT/RR^1/3^
Framingham	QT + 0.154 (1000 − RR)
Hodges	QT + 105 (1/RR − 1)

### 1.2 Inward and outward currents determine the duration of the ventricular APs

**Fig. 1:**
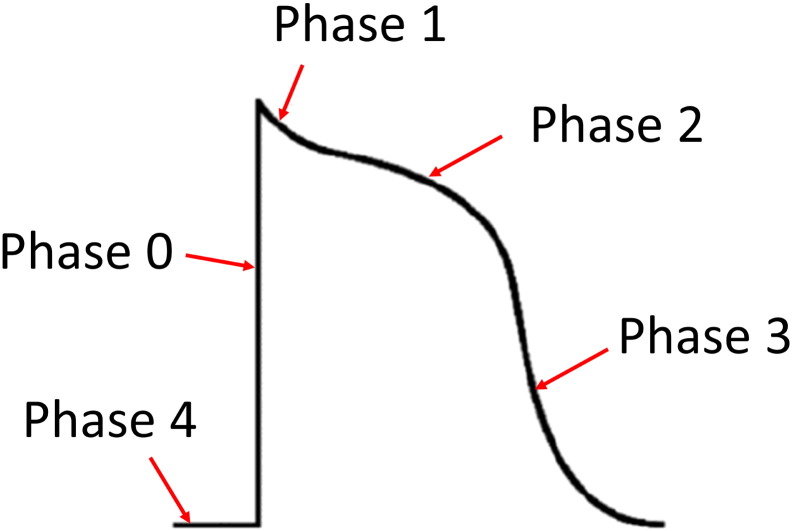
Morphology of the human ventricular action potential. Phase 0 is the action potential upstroke mediated by Na^+^ channel activation. Phase 1 represents early rapid repolarization due to transient outward K^+^ currents. Phase 2 is the plateau phase determined by a balance between inward Ca^2 +^ and outward K^+^ currents. Phase 3 is late repolarization attributed to delayed rectifier K^+^ currents, bringing the membrane potential back to the baseline (phase 4).

Generation of the ventricular APs is dependent upon voltage-gated conductances, and AP durations are determined by the balance between inward and outward currents. An AP has five phases: fast upstroke (phase 0) followed by a spike (phase 1) and plateau (phase 2) morphology, and further repolarization (phase 3), where the transmembrane voltage returns to the resting membrane potential (phase 4) (Fig. 1). Phase 0 is mediated by voltage-gated Na^+^ channels with rapid activation and inactivation kinetics. Phase 1 involves rapid repolarization mediated by the fast and slow transient outward K^+^ currents, *I*_to,f_ and *I*_to,s_, respectively. Phase 3 is maintained by competing inward currents mediated by the voltage-gated L-type Ca^2 +^ channel (*I*_Ca,L_) and Na^+^-Ca^2 +^ exchanger (*I*_NCX_), and outward currents mediated by the voltage-gated delayed rectifier K^+^ channels (*I*_K_) [10]. Phase 3 can be explained by a high driving force for K^+^ efflux due to a large potential difference between the membrane potential and the K^+^ equilibrium potential. Phase 4 is the resting membrane potential at − 80 and − 64 mV [11], [12], [13], which is set by the inward rectifier current, *I*_K1_ with contribution from the weak inward rectifying ATP-dependent K^+^ channels (*I*_K,ATP_) [14]. The QT interval includes the durations of both ventricular depolarization and repolarization. Importantly, the end of repolarization (action potential duration, APD) usually coincides with the resumption of tissue excitability (effective refractory period, ERP).

### 1.3 Long QT syndromes (LQTS)

Long QT syndromes (LQTS) is characterized by an abnormally long QT interval of ≥ 450 ms on the ECG. The first hereditary long QT syndrome was discovered by Jervell and Lange-Nielsen (JLN) in 1957 [15]. In this family, the parents had normal QT intervals and hearing, producing six children. Four suffered from both long QT interval and congenital sensorineural deafness and the remaining two were normal. Three of these four children suffered from sudden death. JLN syndrome was later shown to have an autosomal recessive inheritance. In the 1960s, Romano and Ward separately reported families suffering from QT prolongation but normal hearing, and the syndrome, after whom is named, has an autosomal dominant inheritance [16].

LQTS is caused by a decrease in repolarizing currents or an increase in depolarizing currents, with either congenital or acquired causes. Today, thirteen genetic LQTS subtypes have been identified thus far. Loss-of-function mutations in the different types of K^+^ channels are responsible for LQTS types 1 (KCNQ1), 2 (KCNH2), 5 (KCNE1), 6 (KCNE2), 7 (KCNJ2) and 13 (KCNJ5). By contrast, gain-of-function mutations in Na^+^ channel subunits lead to LQTS types 3 (SCN5A) and 10 (SCN4B), and in the L-type Ca^2 +^ channel produces LQT type 8 (CACNA1C, Timothy syndrome). Mutations in supporting proteins are responsible for the LQT type 4 (ANKB), 9 (CAV3), 11 (AKAP9) and 12 (SNTA1) phenotypes. Recent studies have implicated calmodulin mutations in patients suffering from a LQTS with previously unidentified genetic causes [17], [18], [19]. Moreover, a long QT phenotype has been implicated in sudden unexpected death in epilepsy (SUDEP), caused by increased late Na^+^ current (*I*_Na, L_) mediated by neuronal Na^+^ channel isoforms [20], [21].

**Fig. 2:**
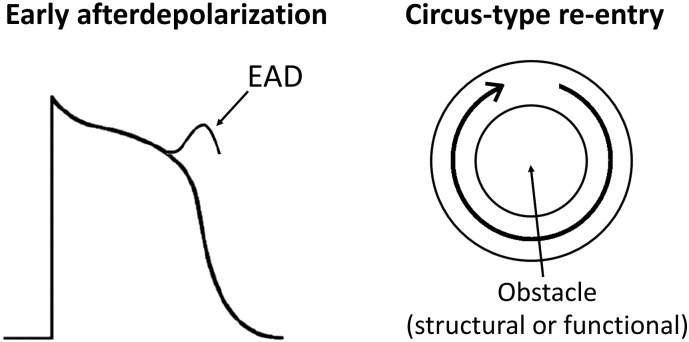
Early afterdepolarizations can produce triggered activity, which can initiate arrhythmias. Circus-type reentry requires slowed conduction, unidirectional conduction block and a central obstacle around which the action potential wave can circulate.

**Fig. 3:**
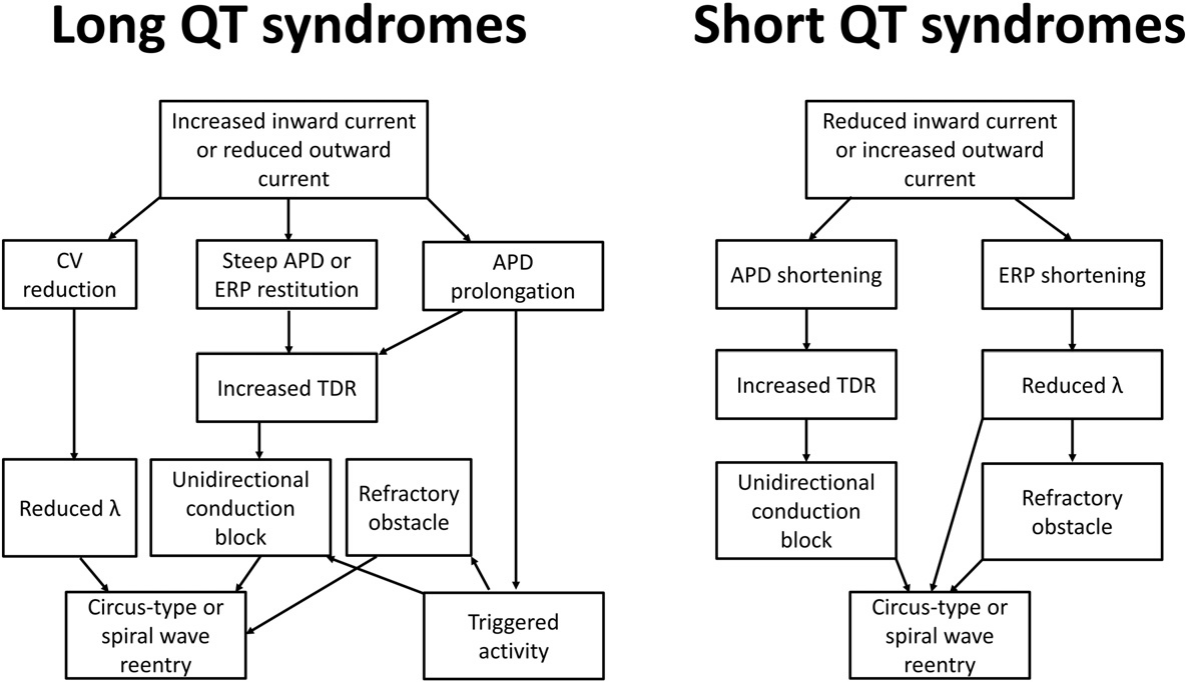
Arrhythmogenic mechanisms in long and short QT syndromes. Prolongation in action potential duration (APD) can predispose to the development of early afterdepolarizations, in turn producing triggered activity that can serve to initiate arrhythmias. Reentrant substrates may involve alterations in conduction velocity (CV), prolongation or shortening of APD or shortening of effective refractory period (ERP). These abnormalities can in turn lead to increased transmural dispersion of repolarization (TDR), which can promote unidirectional conduction block and an obstacle around which the action potential can circulate. Together with reduced wavelength (λ = CV × ERP), these can increase the susceptibility of tachycardia by circus-type or spiral wave reentry.

By contrast, acquired causes of LQTS are much more common than genetic causes. These are commonly due to electrolyte abnormalities, most frequently hypokalaemia. A hypokalaemia mouse model has been used to study the arrhythmogenic mechanisms of LQTS, demonstrating several consequences of APD prolongation (Table 2). Firstly, it increases the Ca^2 +^ current available Na^+^ channel reactivation during the repolarizing phase, leading to the development of early afterdepolarizations and subsequent triggered activity (Fig. 2) [22]. Secondly, AP prolongation preferentially occurs at the epicardium compared to the endocardium, resulting in an increase in the transmural dispersion of repolarization (TDR) [22]. Reduced ERP of the ventricular myocardium [23] and unaltered conduction velocity (CV) were observed, leading to a decrease in excitation wavelength (λ) given by CV x ERP. λ is the path length that is occupied by the action potential wave. Theoretically, a smaller λ can more easily support a re-entrant circuit, thereby increasing the likelihood of reentrant arrhythmias (Fig. 3). In congenital long QT syndromes, the ERP is not typically altered. Similarly, CV is not reduced unless the specific mutation produces loss-of-function mutations in Na^+^ channels, which may give rise to overlapping phenotype of LQTS with Brugada syndrome and conduction defect [24], [25], [26], [27]. Moreover, the emergence of APD alternans, attributed to increased steepness of APD restitution together with the abnormal repolarization gradient can lead to unidirectional conduction block and thereby reentry [28], [29].

**Table 2:** Electrophysiological changes observed in long and short QT syndromes (LQTS and SQTS). CV: conduction velocity. APD: action potential duration. ERP: effective refractory period. TDR: transmural dispersion of repolarization. λ: excitation wavelength (CV × ERP).

Abnormalities	LQTS	SQTS	References
Molecular mechanisms	Increased inward currents or reduced outward currents	Reduced inward currents or reduced outward currents	[15], [30]
Triggered activity	Early afterdepolarizations from LTCC reactivation	Not observed	[86]
Substrates for reentry			
CV	↔/↓	↔/↑	[63]
APD	↑	↓	[53]
ERP	↑	↓	[63]
TDR	↑	↑	[54], [87]
λ	↓	↓	[37], [63]
APD alternans	↑	N/A	[88], [89]
CV restitution	↔	N/A	[29], [90], [91]
APD restitution	↑ gradient	N/A	[29], [92], [93]
VERP restitution	↑ gradient	N/A	[94]

### 1.4 Short QT syndromes (SQTS)

Short QT syndrome (SQTS) is characterized by an abnormally short QT interval of < 350 ms on the ECG. It predisposes affected individuals to an increased risk of atrial and ventricular arrhythmias, in particular ventricular fibrillation, and is therefore an important cause of sudden cardiac death [30]. Shortening of QT interval reflects accelerated repolarization, which can result from increased activity of repolarizing currents, or decreased activity of depolarizing currents. SQTS, like LQTS, can have congenital or acquired causes. Six genetic subtypes of SQTS have been identified thus far. Gain-of-function mutations in the K^+^ channel genes, KCNH2, KCNQ1 [31], [32] and KCNJ2 [33] are responsible for SQT types 1, 2 and 3, respectively. By contrast, loss-of-function mutations in L-type Ca^2 +^ channel subunits, CACNA1C, CACNB2 and CACNA2D1, are found in SQT types 4, 5 and 6, respectively [34]. Interestingly, some patients diagnosed with Brugada syndrome have demonstrated shortened QT intervals [34]. This is perhaps not surprising upon consideration of the molecular mechanisms involved, because loss-of-function mutations in the inward currents, which tips the net current in the outward direction, are observed in both SQTS and Brugada syndrome. Acquired causes are more common, including electrolyte abnormalities of hyperkalaemia or hypercalcaemia, myocardial ischaemia, acidosis or carnitine deficiency [35], [36]. Hyperthermia can also cause a shortened QT interval, as can drugs such as digitalis, acetylcholine, catecholamines or K_ATP_ activators. Short QT intervals have also been associated with epilepsy, particularly during the ictal and post-ictal states [8].

The mechanism of arrhythmogenesis in SQTS is less well-understood than that of LQTS. Recent work in mice demonstrated shortening in ERP in concert with APD [37], leading to decreased λ and a higher risk of circus-type reentry. Abnormal APD restitution leading to APD alternans is unlikely to play a role in SQTS because only long diastolic intervals are engaged where the restitution curve is flat, unlike the case of LQTS where it was possible to engage the steep portion of the restitution curve at low diastolic intervals [28]. CV may be increased due to ERP shortening, but this would not be expected to be pro-arrhythmic since this would increase rather than decrease λ [38]. The similarities and differences of the electrophysiological consequences of LQTS and SQTS are detailed in Table 1.

### 1.5 Arrhythmic risk prediction: markers based on repolarization and QT interval

Pre-clinical models have been useful for the studying the mechanisms of cardiac arrhythmogenesis and provide a platform for testing the arrhythmogenic potential of drugs [29], [37], [39], [40], [41], [42], [43], [44], [45], [46], [47]. Experiments in these systems have demonstrated different arrhythmic risk markers, such as increased TDR given by the maximum APD difference across the myocardial wall [48], increased critical interval for re-excitation given by the APD-ERP difference [23], [49], [50], [51], shortened λ and reduced λ-TRIAD (which is based on λ and repolarization properties of triangulation, reverse use dependence, instability and dispersion: TRIaD) [52]. The clinical marker traditionally used for predicting arrhythmic risk has been QT_c_ [53]. However, its lack of accuracy led to the development of other markers [52], such as QT dispersion (QT_d_) [54], [55], interval from the peak to the end of the T wave [56] (T_peak_ – T_end_, reflecting increased TDR [57]), and (T_peak_ – T_end_)/QT ratio [58].

However, none of the above repolarization markers takes into account action potential conduction, yet λ, which incorporates both processes, is an important determinant of arrhythmic risk [59], [60], [61]. Three novel markers based on conduction-repolarization have been proposed thus far. The first is the index of Cardiac Electrophysiological Balance (iCEB: QT/QRS) proposed by Lu and colleagues, which is a surrogate marker of λ [62]. Indeed, this has demonstrated utility in predicting arrhythmic risk in drug-induced settings, LQTS and Brugada syndrome [63]. The other two markers, (T_peak_–T_end_)/QRS and (T_peak_–T_end_)/(QT × QRS), have recently been put forward by Tse, but these remain to be validated clinically. Tse's indices are based on the observations that both conduction and repolarization abnormalities are important in arrhythmogenesis and T_peak_–T_end_ was a significant predictor of SCD even after adjusting for, *inter alia*, QT_c_ and QRS durations [64]. They may therefore provide superior predictive values for arrhythmic risk than the repolarization markers discussed above and even iCEB [65], [66]. Tse's indices were subsequently modified by Tse and Yan to incorporate QRS dispersion (QRS_d_) reflecting CV dispersion, yielding QRS_d_ × (T_peak_–T_end_)/QRS, and QRS_d_ × (T_peak_–T_end_)/(QRS × QT) [67]. It was proposed that the term QRS_d_/QRS could be a surrogate marker of CV dispersion coefficient based on the standard deviation of the mean CV [68]. Other non-invasive methods of assessing the function of the heart include magnetocardiography, which may provide additional insights into risk stratification in the future [69], [70], [71], [72], [73], [74], [75], [76].

### 1.6 Therapeutic strategies

**Fig. 4:**
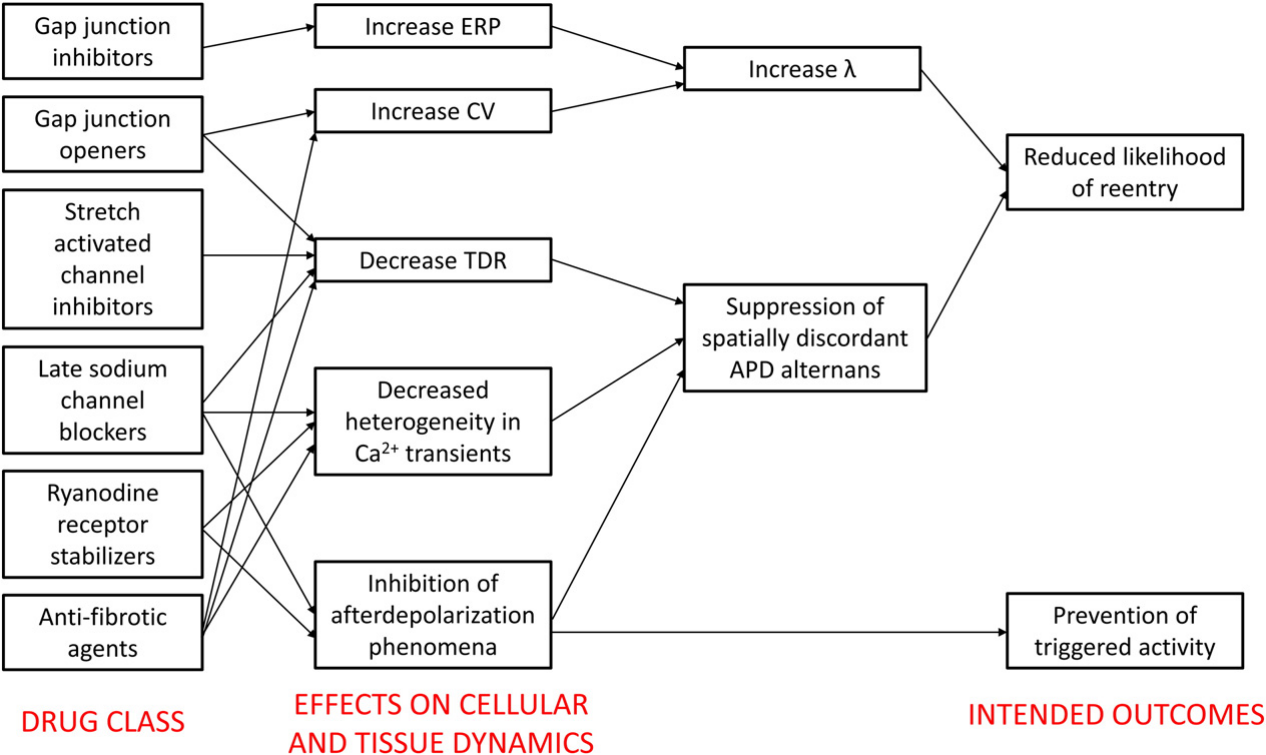
Future drug classes for anti-arrhythmic therapy based on rational drug design: gap junction inhibitors, gap junction openers, stretch-activated channel inhibitors, late sodium channel blockers, ryanodine receptor stabilizers and anti-fibrotic agents. Adapted from Tse et al. (2016) with permission [28].

For LQTS, beta blockers are only effective in preventing ventricular tachycardia in approximately 70% of the patients. The remaining 30% are susceptible to arrhythmias. For SQTS, quinidine or disopyramide are recommended. In both syndromes, definitive treatment is implantable cardioverter-defibrillator (ICD) insertion. There is therefore a need to develop more effective agents for anti-arrhythmic therapy. A better understanding of the mechanisms of arrhythmogenesis would allow rational drug design that aims to reverse the electrophysiological abnormalities in question. Application of pre-clinical results to clinical medicine could result in effective translation for the benefit of patients, which is illustrated by the following two examples that demonstrate important proofs-of-concept. Firstly, hypokalaemia modelling LQTS produces AP prolongation, reduced ERP, reduced λ, increased TDR, increased APD restitution slopes and increased amplitude of APD alternans. Gap junction inhibition using heptanol normalized ERP and therefore λ without correcting for the remaining repolarization abnormalities [29]. Secondly, hyperkalaemia modelling SQTS results in shortened APD and ERP, reduced λ and increased TDR. Anti-arrhythmic effects of hypercalcaemia were associated with reversal of ERP changes and normalization of λ, again without correcting for the repolarization abnormalities [37]. Together, the above studies demonstrate that prolonging myocardial refractoriness with an aim of increasing λ is a viable strategy. Other approaches that have demonstrated some success in pre-clinical models are increasing ERP or CV, decreasing heterogeneities in CV, APD, ERP or Ca^2 +^ transients, or suppressing after depolarization phenomena (Fig. 4: modified from Tse et al. with permission [28]). Novel agents using such strategies are gap junction inhibitors [77], [78], [79], [80] and openers [81], [82], stretch-activated channel modulators, late sodium channel blockers [83], ryanodine receptor stabilizers [84] and anti-fibrotic agents [85]. It is likely that a systems physiology approach will play a large role in studying the complex spatial and temporal properties of cardiac dynamics. Its application will no doubt transform arrhythmia management by identifying agents that have lower toxicity and toxic side effects of currently available drugs.

## Conflict of interest

None declared.
